# Fractionation of muscle activity in rapid responses to startling cues

**DOI:** 10.1152/jn.01009.2015

**Published:** 2016-12-21

**Authors:** Lauren R. Dean, Stuart N. Baker

**Affiliations:** Institute of Neuroscience, Newcastle University, Newcastle upon Tyne, United Kingdom

**Keywords:** startle, reticulospinal tract, fractionation, StartReact

## Abstract

Movements in response to acoustically startling cues have shorter reaction times than those following less intense sounds; this is known as the StartReact effect. The neural underpinnings for StartReact are unclear. One possibility is that startling cues preferentially invoke the reticulospinal tract to convey motor commands to spinal motoneurons. Reticulospinal outputs are highly divergent, controlling large groups of muscles in synergistic patterns. By contrast the dominant pathway in primate voluntary movement is the corticospinal tract, which can access small groups of muscles selectively. We therefore hypothesized that StartReact responses would be less fractionated than standard voluntary reactions. Electromyogram recordings were made from 15 muscles in 10 healthy human subjects as they carried out 32 varied movements with the right forelimb in response to startling and nonstartling auditory cues. Movements were chosen to elicit a wide range of muscle activations. Multidimensional muscle activity patterns were calculated at delays from 0 to 100 ms after the onset of muscle activity and subjected to principal component analysis to assess fractionation. In all cases, a similar proportion of the total variance could be explained by a reduced number of principal components for the startling and the nonstartling cue. Muscle activity patterns for a given task were very similar in response to startling and nonstartling cues. This suggests that movements produced in the StartReact paradigm rely on similar contributions from different descending pathways as those following voluntary responses to nonstartling cues.

**NEW & NOTEWORTHY** We demonstrate that the ability to activate muscles selectively is preserved during the very rapid reactions produced following a startling cue. This suggests that the contributions from different descending pathways are comparable between these rapid reactions and more typical voluntary movements.

## Abstract (precis)

We demonstrate that the ability to activate muscles selectively is preserved during the very rapid reactions produced following a startling cue. This suggests that the contributions from different descending pathways are comparable between these rapid reactions and more typical voluntary movements.

the acoustic startle response is a stereotyped contraction of muscles throughout the body in response to an unexpected loud sound (Li et al. 2001) and is believed to represent a mechanism for protecting vulnerable parts of the body from attack by predators. In humans, the temporal sequence of muscle activation is consistent with acoustic startle originating within the brainstem (Brown et al. 1991). Analysis in rodents has revealed a key role for the reticulospinal tract in mediating startle. After a stimulus is received via the cochlea, it is relayed through the ventral cochlear nucleus (Davis et al. 1982) or cochlear root neurons to the caudal pontine nucleus (PnC) of the reticular formation (Wu et al. 1988). Lesions of the PnC lead to abolition of the startle response (Hammond 1973).

A distinct but related phenomenon is the ability of acoustic startling stimuli to accelerate a voluntary reaction compared with that seen after a less intense cue. This is referred to as the “StartReact” effect (Valls-Solé et al. 1995). The mechanism underlying StartReact is subject to some debate. The shortened reaction times appear too rapid to involve purely cortical pathways, given known conduction delays to the cortex and from the cortex to the arm (Carlsen et al. 2004a). This leads to the suggestion that subcortical mechanisms may play a role, and two potential mechanisms have been proposed (Carlsen et al. 2011). One possibility is that a voluntary motor program is prepared and then stored in subcortical structures before being triggered by the loud cue without cortical involvement (Carlsen et al. 2004a). Alternatively, the startling cue might simply generate a diffuse activation, which raises motoneurons closer to threshold. Upon this is superimposed the usual motor output associated with a voluntary movement, which is thereby able to produce overt output earlier because of the preceding input to motoneurons.

Against this latter idea, differences in reaction time following different cue modalities are not preserved when startle cues are added (Carlsen et al. 2011). In addition, choice reactions where different movements must be made in response to different cues do not exhibit a StartReact effect (Carlsen et al. 2004a). It appears necessary for the movement to be preprepared, supporting the idea of subcortical structures acting as a “holding pen” for the prepared movement, which once triggered can then unfold without cortical involvement (Carlsen et al. 2007).

Given the importance of the reticular formation in startle, it is a good candidate for the postulated subcortical structure involved in StartReact. In support of this notion, StartReact responses are reduced in patients with gait freezing in Parkinson’s disease, which is associated with dysfunction of the reticular formation and pedunculopontine nucleus (Nonnekes et al. 2014). StartReact responses are greater in patients with corticospinal tract degeneration but no clinical weakness, which would be consistent with strengthened reticulospinal connections partially compensating for corticospinal loss (Fisher et al. 2013). StartReact acceleration of reaction time is not seen in isolated movements of the index finger (Carlsen et al. 2009), which may be consistent with a presumed proximal bias of the reticulospinal tract (but see Baker 2011; Kuypers et al. 1960). However, StartReact effects can be observed if subjects respond with a precision grip (Honeycutt et al. 2013), which is consistent with recent data showing reticulospinal connections to muscles acting on the hand (Riddle and Baker 2010; Riddle et al. 2009) and modulation of reticular formation cells with finger movements (Soteropoulos et al. 2012).

The reticulospinal tract differs in several respects from the corticospinal tract, which forms the major substrate for voluntary movement in primates. Importantly, corticospinal axons terminate in small numbers of motoneuron pools (Buys et al. 1986; Shinoda et al. 1981), allowing fractionated activation of muscles to produce fine movements. By contrast, reticulospinal axons diverge extensively in the spinal cord (Matsuyama et al. 1999; Matsuyama et al. 1997; Peterson et al. 1975). In stroke patients who are presumed to rely mainly on reticulospinal outputs following damage to the corticospinal tract, muscles often show obligate coupling of activity which can impair functional movements (Dewald et al. 1995; Lang and Schieber 2004). If it is true that StartReact responses rely more on the reticulospinal tract, we would expect that such responses would show less fractionation than standard voluntary reactions.

In this study, we used a method previously applied by Weiss and Flanders (2004) to quantify movement fractionation. Subjects performed a wide variety of motor tasks in response to startling or nonstartling auditory cues, while muscle activation patterns were measured using electromyogram. We show that similar fractination patterns were generated for movements following both types of cue. This suggests that the relative contributions of different descending pathways are similar for StartReact and movements made in response to nonstartling cues.

## METHODS

### Subjects

Ten human volunteers (5 males, 5 females; mean age ± SD: 43 ± 13 yr) with no known neurological deficits participated in this experiment. All subjects were right-handed as evaluated through self-report. All procedures were approved by the Newcastle University Medical Faculty Human Subject Ethics Committee, and subjects provided written informed consent.

#### Task.

**Fig. 1.**
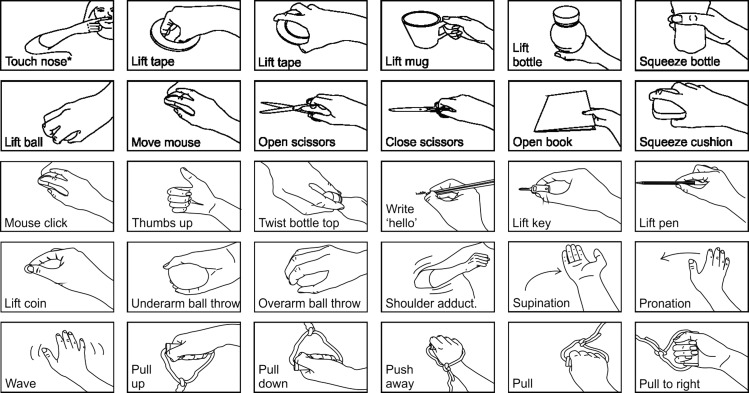
The 32 movement tasks subjects were asked to perform. The touch nose task (*) was performed with 3 different starting positions: with the arm outstretched above the head, with the arm outstretched to the right, and with the arm hanging by the side of the body.

Subjects were asked to carry out 32 different motor actions as quickly as possible after an auditory cue while comfortably seated at a table. The tasks were selected to elicit a wide range of activation patterns in muscles acting across shoulder, elbow, wrist, and finger joints (Fig. 1). The experimenter first demonstrated how to carry out each movement; consistent performance was checked in initial practice trials. Recordings were made when subjects made the instructed movement in response either to a quiet (80 dB, 500 Hz, 10 ms) or loud beep (115dB; this level being the loudest subjects could tolerate over the many repetitions used) delivered via headphones. The intensity of the loud beep was sufficient to evoke an overt startle response in subjects when they first experienced it. Movements were carried out in blocks of 10, five in response to each cue, with intertrial interval ranging between 4.2 and 7.5 s to prevent anticipation of cue timing. Four different random sequences of loud/quiet cues were used to prevent subjects predicting forthcoming cues; the sequence used for a given task was also determined randomly.

#### Electromyography recording.

EMG was recorded from 15 muscles using bipolar surface electrodes (Bio-Logic M0476; Natus Medical, Mudelein, IL) in a belly-tendon montage and secured with Micropore tape (3M, St. Paul, MN). Muscles recorded from included the first dorsal interosseous (1DI), abductor pollicis brevis (AbPB), abductor digiti minimi (AbDM), flexor digitorum superficialis (FDS), flexor carpi ulnaris (FCU), flexor carpi radialis (FCR), extensor carpi ulnaris (ECU), extensor carpi radialis (ECR), extensor digitorum communis (EDC), brachialis (BR), biceps (BIC), triceps (TRI), anterior deltoid, posterior deltoid, and pectoralis major. Muscles were identified by palpation while asking subjects to perform hand and arm movements designed to activated the desired muscle. The neck muscle sternocleidomastoid (SCM) was also recorded in most subjects. A ground electrode was positioned on the dorsum of the right hand. EMG signals were amplified by D360 isolated amplifiers (Digitimer, Welwyn Garden City, UK), notch filtered (49–51 Hz) to remove mains frequency contamination, band-pass filtered (30 Hz-2 kHz), and digitized at a sampling rate of 5 kHz using a micro1401 data capture system connected to a PC running Spike2 software (Cambridge Electronic Devices, Cambridge, UK).

### Analysis

**Fig. 2.**
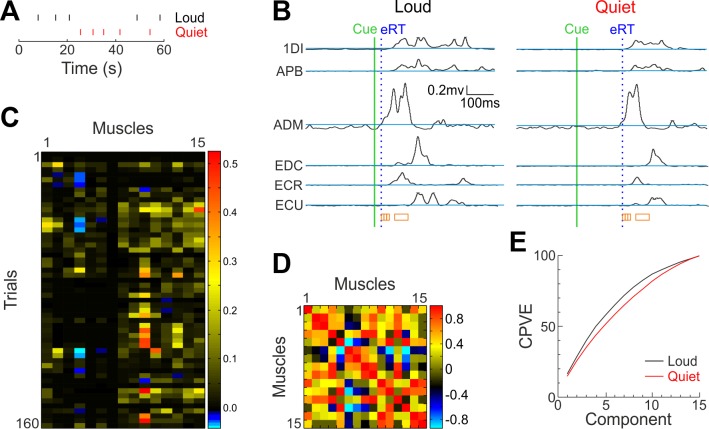
The analysis procedure. *A* and *B*: first, data surrounding the stimulus cue (green line) were retrieved. Reaction time (RT) threshold was defined for each muscle as 3 SD above mean baseline (blue lines), and RTs were determined as the first point where traces exceeded this threshold. The earliest RT (eRT) across muscles for each trial was found (blue dotted line), after which four different windows of EMG (0–10, 10–20, 20–30, and 50–100 ms) were taken (orange boxes). The mean across each window for each muscle was calculated for each trial and added to a matrix of muscles × trials (heat map, *C*). At this point, any loud trial with an eRT of >100 ms was removed, along with the same number of randomly selected quiet trials for each subject. All analysis following this stage therefore used the reduced trial number. After Z-score standardization, the covariance matrix of this matrix was calculated (heat map, *D*) and principal component analysis (PCA) was carried out on this. PCA yielded a number of components, each of which explained a percentage of the variance in the EMG data represented by the matrix shown in *C*. Components were in descending order of the percentage variance explained (PVE). The cumulative sum of the PVE was calculated (CPVE; *E*). 1DI, first dorsal interosseous; APB, abductor pollicis brevis; ECU, extensor carpi ulnaris; ECR, extensor carpi radialis; EDC, extensor digitorum communis; ADM, abductor digiti minimi.

EMG recordings were analyzed using custom scripts in Matlab (The Mathworks, Natick, MA). EMG was first rectified, and a threshold was defined for each muscle as 3 SD above the mean of the 200-ms section of baseline EMG preceding the stimulus marker. The reaction time for a given muscle and trial was defined as the first time after the stimulus marker that the EMG exceeded this threshold. All traces were visually inspected; any assigned reaction time that was inaccurately placed by the automated script (e.g., due to an artifact) was manually corrected. For a given trial, the earliest reaction time (eRT) across all muscles was used as a temporal reference point. Averages of rectified EMG were extracted for each muscle in time windows 0–10, 10–20, 20–30, and 50–100 ms after this point (Fig. 2*B*, orange squares). For each window timing, this led to 32 matrices (1 per task type) with 5 rows (corresponding to the number of trials per task) and 15 columns (corresponding to the number of muscles) for each of the loud-cued and for quiet-cued movements.

To ensure that only trials where a StartReact effect was seen were included, any trials following the loud cue which had an eRT longer than 100 ms were excluded. To avoid using an unequal number of trials between conditions, the same number of trials following the quiet cue was also excluded (selected at random). The number of trials removed in this process ranged across subjects from 28 to 141 trials per cue type. The muscle activation matrices were then combined across the 32 different tasks, resulting in an *n* × 15 matrix for each subject (Fig. 2*C*), where *n* represents 160 (32 tasks × 5 trials per task) minus the number of trials excluded. Activities were normalized so that each muscle’s values had zero mean and unit SD.

From the normalized activity matrix we found the covariance between each muscle pair (Fig. 2*D*); eigen decomposition of this matrix yielded the principal components (the eigen vectors) and the proportion of variance explained by each component (the eigen values). Eigen values were sorted, and their cumulative sum calculated to determine the cumulative percentage of variance explained (CPVE; Fig. 2*E*). This indicated the percentage of total variance in muscle activity patterns that could be explained by simplifying the data to a reduced number of dimensions. A highly fractionated pattern of muscle activity would have a curve that rose slowly as the number of components increased, indicating a complexity that was only poorly represented by dimensional reduction. By contrast, a curve that rose close to 100% with only a small number of components would be indicative of a simple, stereotyped pattern of muscle activity.

One problem with this method for determining CPVE is that for some subjects and tasks, quiet cue trials had to be excluded at random to ensure that equal numbers of loud and quiet cue trials were considered. To ensure that results were representative, the analysis was repeated 100 times for each subject (with different random selections of quiet cue trials), and the CPVE curves used for that individual were formed from the average of the CPVE curves determined for each run.

**Fig. 3.**
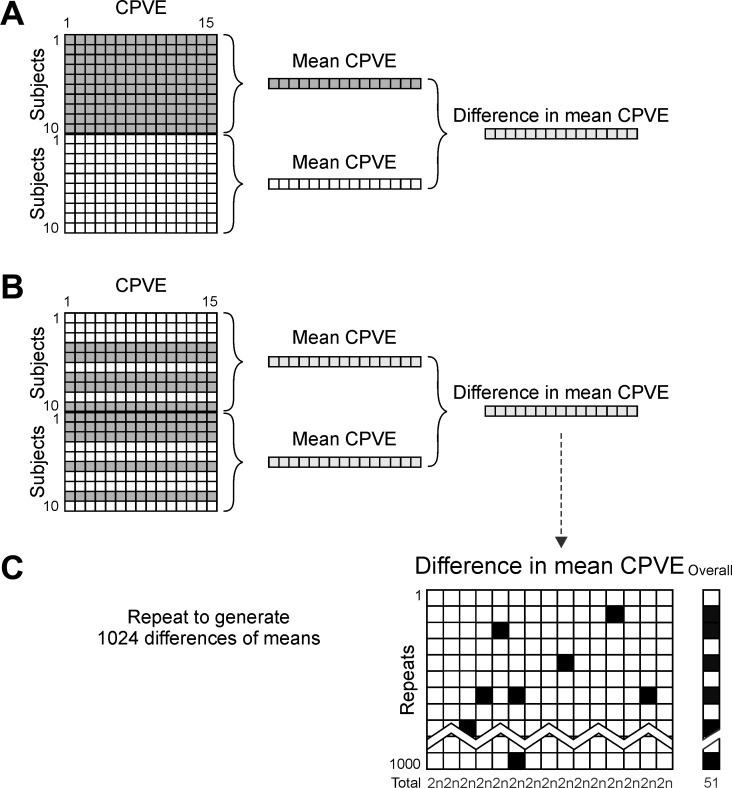
Flow chart of Monte Carlo simulation for significance testing of difference in mean CPVE for each component. Grey boxes represent loud cued trials, white boxes represent quiet cued trials. First, the mean of each of the loud and quiet cued original matrices was taken and the difference between these found (*A*). The original loud and quiet cued matrices were then shuffled within subjects and the difference of the means of the shuffled matrices calculated (*B*). This shuffling and averaging process was repeated 1,024 times for each time window. This resulted in a final matrix of differences in mean CPVE between shuffled conditions (*C*). Those elements that were in the most extreme 2*n* out of 1,024 in each column were marked as significant (black). Each shuffle was marked as significant if one or more element was significant (black on *far right*). The detection threshold for an individual measure (*n*) was chosen to provide an overall significance limit of *P* < 0.05, corresponding to 51/1,024 false positive detections (see counts at the *bottom* of each column).

To compare fractionation in StartReact vs. standard reactions, values of CPVE were averaged across subjects separately for the loud and quiet cues. Statistical comparison of these curves is complex, as the values are not independent: for example, the CPVE for component 3 is equal to the CPVE for component 2 plus the percentage of variance explained by component 3 alone. Standard approaches to correct for multiple comparisons are therefore not appropriate. We instead used a Monte Carlo approach to determine whether there were significant differences. First, the difference in mean CPVE for loud and quiet cues was determined (Fig. 3*A*). Then, CPVE curves were shuffled for each subject, mean CPVE curves calculated, and differences found (Fig. 3*B*). This process was repeated 2^10^ = 1,024 times, yielding all possible shuffles over 10 subjects. These 1,024 differences in mean CPVE curves were distributed on the null hypothesis that there was no difference in CPVE between quiet and loud cues (Fig. 3*C*). Mean CPVE values were sorted, and threshold values chosen as the value ranked *n*’th and (1,024-*n*)’th, where *n* was a parameter determined as below.

The overall false positive rate was determined by counting how many of the 1,024 surrogate curves of CPVE difference had at least one component outside these threshold values. The value of *n* was then adjusted to be the highest value, which produced an overall false positive rate less or equal to 51/1,024, corresponding to *P* ≤ 0.05 (Fig. 3*C*, column labeled “overall”). Finally, the experimental curve of difference in mean CPVE (Fig. 3*A*) was compared with the thresholds determined from the surrogates; any values above or below the thresholds were marked as significantly different. If one or more component out of the 15 available was different on this test, we concluded that there was a significant difference between the CPVE following loud and quiet cues with *P* < 0.05.

The above procedure was carried out separately for all time windows.

A Wilcoxon rank sum test was used to compare eRTs across muscles for every trial, task, and subject between quiet and loud conditions, as they were not found to be normally distributed as assessed by a Kolmogorov-Smirnov test.

As an alternative approach to investigate fractionation of muscle activation during the performance of the different tasks, we also analyzed the similarity of the muscle activity patterns. If the mean rectified EMG over a particular time window relative to the eRT in the 15 available muscles is represented as the 15-dimensional vector ***x_j_***, then the similarity between trials *j* and *k* was calculated as

$${\text{Similarity}}_{j,k}=\frac{x_{j}.x_{k}}{\left|x_{j}\right|\left|x_{k}\right|}$$

where ***x_j_***.***x_k_*** denotes the vector dot product and |·| denotes the vector magnitude. This definition of similarity is just the cosine of the angle between the vectors and will be equal to one if the vectors point in the same direction or zero if they are orthogonal.

Four different sets of similarity calculations were performed. The first found the similarity between each trial of a given task with a quiet cue, with all other trials of the same task. A second measure did the same but following a loud cue. Third, we calculated the similarity between each trial of a given task and all trials of a different task for responses to a quiet cue. Finally, we calculated the similarity between each trial of a given task following a loud cue, with each trial of the same task following a quiet cue. The cumulative distribution of these three measures was plotted, combining each set of measurements across all pairs of trials, the 32 tasks and 10 subjects. This allowed us to see whether muscles were activated in a similarly selective pattern appropriate for a given task after loud and quiet cues.

## RESULTS

### Reaction Times

**Fig. 4.**
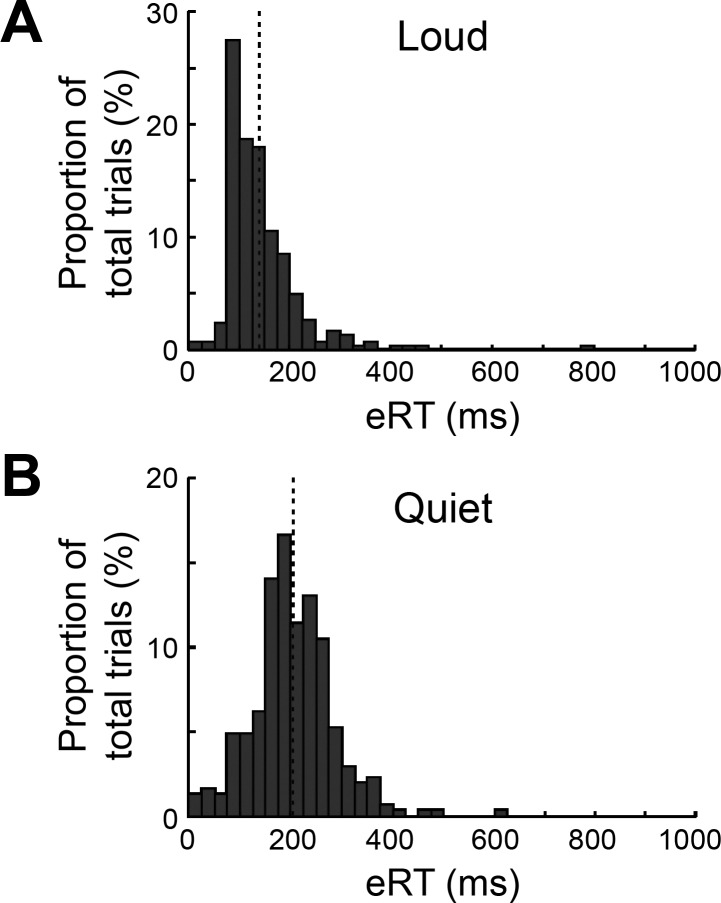
Histograms of eRTs. Each trial has contributed 1 count to these plots; results are combined across subjects, movement type, and repeats of each movement but presented separately for loud and quiet cues. The dotted lines represent the mean in each case. All trials contributed data to this figure, including those with long eRTs following loud cues, which were excluded from the main analysis.

The mean earliest RT across muscles for each trial, task and subject for the quiet condition was 204 ± 79 ms (means ± SD) and for the loud condition was 144 ± 75 ms (Fig. 4). The difference in RT between conditions was significant (*P* < 0.00001, Wilcoxon rank sum test). The difference between the mean of these early RTs for each condition was 60 ms; this considerable reaction time shortening, comparable to previous work, provided confidence that the Startreact phenomenon had been evoked in our setup.

### Principal Component Analysis

**Fig. 5.**
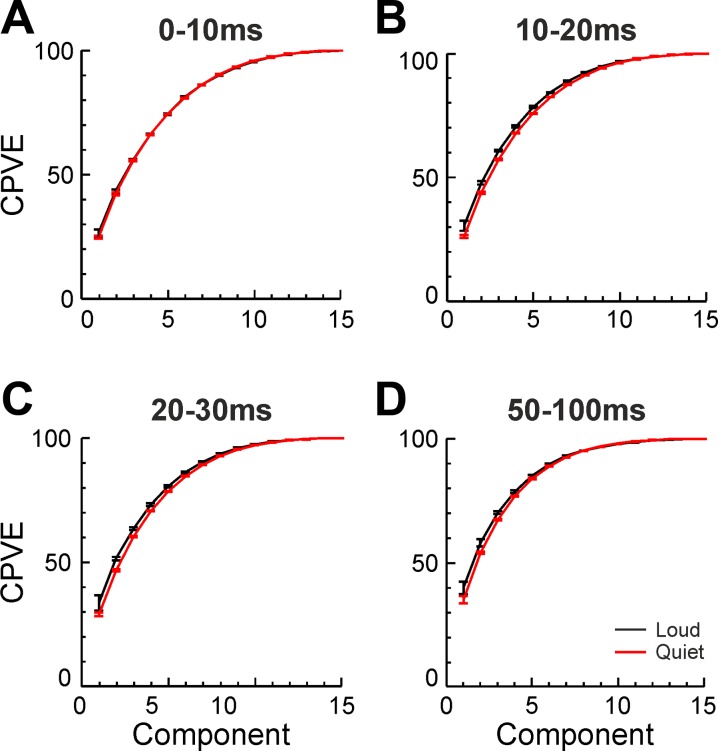
The mean CPVE for both loud (black) and quiet (red) cued trials, as found by PCA carried out on mean EMG from 4 different time windows (*A*–*D*) relative to the eRT. There was no significant difference between results with loud and quiet cues for any of the time windows (*P* > 0.05). Error bars represent 1 SE of the mean above and below the mean.

Principal component analysis was carried out on the mean of each of four different time periods of the EMG recording (0–10, 10–20, 20–30, and 50–100 ms after earliest reaction time, Fig. 5).

There were no significant differences between the CPVE of loud and quiet trials for any time period, as assessed by Monte Carlo analysis. We therefore accept the null hypothesis that the loud and quiet cue trials have the same distribution of CPVE, with *P* > 0.05. As a null result, it is of course possible that there were differences but that these were too small to be detected using the number of subjects available to us. We explored this by carrying out further analysis using data simulated to have similar mean and variance of CPVE as in the experimental data and then subjected to exactly the same statistical methods. This showed that an average difference in CPVE of one component of >5.5% would be detectable at *P* < 0.05 with 90% power. We can have confidence, therefore, that if any differences in CPVE do exist between loud and quiet cued trials, they are smaller than this value.

### Similarity Analysis

**Fig. 6.**
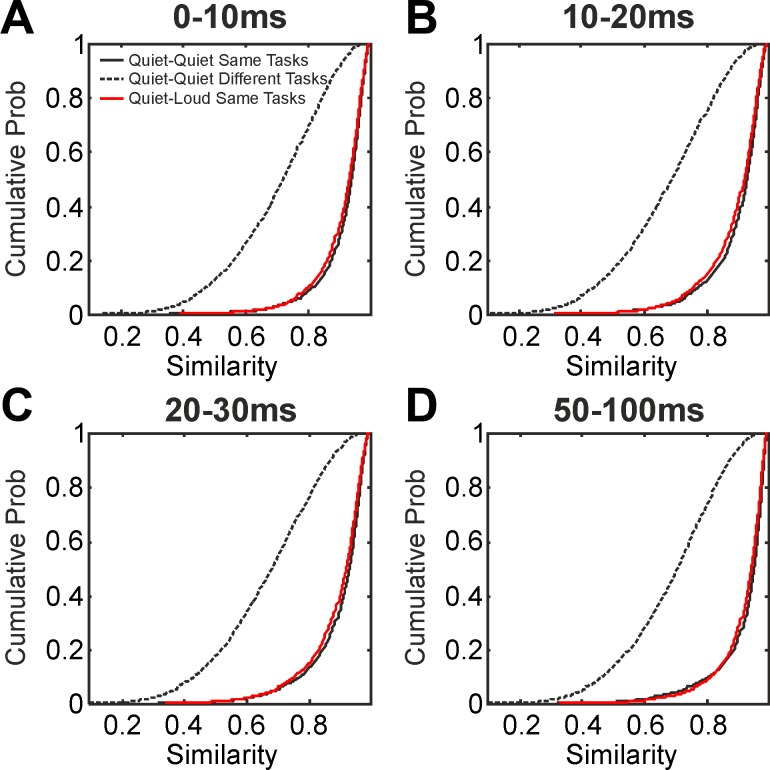
*A*–*D*: the distribution of similarity of muscle activation patterns in 4 time periods relative to the eRT. Solid black line represents the similarity of trials following quiet cues compared with other trials of the same task following a quiet cue. Dotted black line represents the similarity of trials from 2 different tasks, both following a quiet cue. Red lines show the similarity of trials from the same task, 1 following a quiet cue, 1 following a loud cue. In all cases, similarity values are shown as a cumulative probability distribution. The similarity of trials from the same task following a loud cue was computed, but is not shown on these plots for clarity; its distribution was very close to the red lines.

Figure 6 shows the distribution of the similarity of muscle activity patterns (calculated as defined in methods). This analysis was carried out for the same four different time periods relative to eRT as previously (0–10, 10–20, 20–30, and 50–100 ms, Fig. 6, *A*–*D*). In each panel, the black line shows the distribution of similarity (as a cumulative probability plot) when comparing different trials of the same task, in response to a quiet cue. For Fig. 6*A*, 92% of similarity values were above 0.8; similarly high values were seen in other time periods. The dotted black lines show the distribution when comparing trials of different tasks, both in response to a quiet cue. For the early time period shown in Fig. 6*A*, 69% of similarity values were below 0.8. These distributions indicate that there was high consistency in the pattern of muscle activation from trial to trial within a particular task but considerable heterogeneity across tasks.

If muscles are well fractionated in the rapid responses after a loud cue, we would expect the muscle activity patterns to be appropriate for the specific task to be carried out. The red lines in Fig. 6 show the distributions of similarity when comparing trials of the same task following loud cues with those after quiet cues. It will be apparent that the red lines closely overlie the black; the responses after loud cues were almost as similar to those after quiet cues as the quiet cued trials were to each other. In fact, there was a very slight difference in mean similarity for the first three time periods shown, which reached significance given the large number of pairwise similarity measures available (see Table 1). For clarity of display, the distributions of similarity for trials of the same task following loud cues are not shown in Fig. 6; in all cases they were very similar to the red lines (same task, quiet compared with loud cues), and the mean similarity was not significantly different between these two measures (Table 1).

**Table 1.** Measures of average similarity for different time periods after the eRT, computed from the cumulative distribution plots in Fig. 6

Time Period After eRT	Average Similarity Between Trials of the Same Task, Both Quiet Cues	Average Similarity Between Trials of Different Tasks, Both Quiet Cues	Average Similarity Between Trials of the Same Task, Both Loud Cues	Average Similarity Between Trials of the Same Task, Quiet Cues Compared with Loud Cues
0–10 ms	0.913	0.700†	0.908*	0.906†
10–20 ms	0.901	0.673†	0.895*	0.892†
20–30 ms	0.898	0.665†	0.891*	0.889†
50–100 ms	0.921	0.687*†	0.921	0.918

Similarity measures in the 3rd and 4th columns were compared and did not differ significantly (*P* > 0.05). eRT, earliest reaction time.

* *P* < 0.05.

† *P* < 10^−4^, significantly different (*t*-test) from the similarity between trials of the same task following quiet cues (1st column).

## DISCUSSION

The corticospinal tract is likely to be the predominant pathway underlying primate voluntary movement. Given the known high level of fractionation of the corticospinal tract, we therefore expected a slowly rising CPVE curve for movements generated following nonstartling cues. We hypothesized that a markedly less fractionated pattern after startling cues might demonstrate an increased contribution from another pathway such as the reticulospinal tract.

Our results showed a close agreement in the CPVE curves for movements in response to startling and nonstartling cues; this was the case even for the earliest muscle activation following the cue onset. Furthermore, the specific patterns of muscle activity produced for a given task following a loud cue were very similar to those produced for the same task following a quiet cue. This suggests that the different descending pathways, with their different capabilities to activate muscles selectively, contribute similarly to both types of responses considered here. It is known that identified corticospinal neurons modulate their discharge shortly after a cue and around 100 ms before the onset of EMG in a voluntarily-activated muscle (Evarts 1966). These timings would allow a corticospinal tract contribution even to the accelerated responses of StartReact. Conversely, during self-paced finger movements cells in the reticular formation modulate their firing at least as much as those in the hand representation of contralateral motor cortex, suggesting that the reticulospinal tract also plays a role in voluntary movements (Soteropoulos et al. 2012). It is most likely therefore that all movements are generated by a coordinated action from multiple descending systems. The present results indicate that even for the accelerated responses following a startling cue, there is a similar level of control over which muscles are activated as for slower voluntary reactions.

Although there were closely overlapping distributions of similarity between pairs of trials of the same task cued by a quiet sound, and pairs where one trial followed quiet and one loud sounds (Fig. 6), there were significant differences between the means of these distributions (Table 1). Similar small reductions in similarity were also seen when comparing pairs of trials using the same task following a loud cue. Such tiny differences (<1%) were only able to reach significance because of the highly sensitive nature of this analysis, which examined large number of trial pairs, and this difference is likely to have negligible importance in performing the tasks. Nevertheless, this result may provide some evidence for a small shift in the balance of the contributions from the different descending tracts to movement initiation following the loud vs. quiet cued trials.

The earliest work on StartReact recorded from the SCM muscle in the neck, and found that SCM activation was present on all trials with a loud cue, but none with quiet cue (Valls-Solé et al. 1999). Subsequent work found that SCM activation following loud cues was variable and suggested selecting out only those trials with SCM activity to represent genuine StartReact trials (Honeycutt et al. 2013). We did record from the SCM muscle but found it an unreliable marker: in some subjects, SCM activation was often seen following quiet cues. This may reflect the need for neck stabilization in some of our tasks, which had an unconstrained posture. SCM activity is a good marker for an overt startle response, but the StartReact phenomenon has important differences from startle. StartReact does not habituate and does not show prepulse inhibition (Valls-Solé et al. 2005). SCM activity does not habituate during a StartReact experiment; this may indicate that it continues to be a good marker for trials where startle mechanisms have been evoked, but equally this may reflect less specifc requirements for neck stabilization. We therefore preferred to select trials following a loud cue on the basis of their early reaction time, rather than use recordings from this neck muscle which has an unclear relation to the StartReact effect. The clear shift to shorter reaction times, including many less than 100 ms (Fig. 4), provides confidence that a StartReact process was indeed involved.

Responses must be prepared to see a StartReact effect (Carlsen et al. 2004b). Early work on motor preparation focused on cells in premotor cortex. These modulate their discharge during an instructed delay period, in which a movement is prepared but withheld until a “go” cue (Tanji and Evarts 1976; Weinrich et al. 1984). However, subsequent studies reported delay period activation of neurons in the reticular formation (Buford and Davidson 2004), opening the possibility that responses could be stored in the brainstem for subsequent rapid execution during StartReact (Carlsen et al. 2011). Spinal cord interneurons also show preparatory discharge (Prut and Fetz 1999). Since many such interneurons receive convergent input from both corticospinal tract and reticulospinal tract (Riddle and Baker 2010), it is equally possible that a motor program is stored in the spinal cord and triggered by descending pathways. More recent concepts of motor preparation see it as positioning the activity of many neurons in a high-dimensional state space ready to initiate an orderly trajectory corresponding to the required movement (Shenoy et al. 2011). While to date such work has mainly focused on the cortex, the concept could straightforwardly be extended also to embrace subcortical networks. Our finding of similar fractionation for movements after startling and nonstartling cues suggests that these push the motor system along the same preprepared trajectory but that passage along the trajectory toward movement onset is simply speeded up for the loud cue. Importantly, there does not seem to be any trade-off here between speed and accuracy, as commonly seen in other systems and expressed by Fitts’ law (Bertucco et al. 2013)–the rapid movements made following a startling cue are just as well fractionated as slower reactions.

## GRANTS

This work was supported by the Medical Research Council and the Wellcome Trust.

## DISCLOSURES

No conflicts of interest, financial or otherwise, are declared by the author(s).

## AUTHOR CONTRIBUTIONS

S.N.B. conceived and designed research; L.R.D. performed experiments; L.R.D. analyzed data; L.R.D. and S.N.B. interpreted results of experiments; L.R.D. prepared figures; L.R.D. and S.N.B. drafted manuscript; L.R.D. and S.N.B. edited and revised manuscript; L.R.D. and S.N.B. approved final version of manuscript.
